# Pressure and Temperature Sensors Using Two Spin Crossover Materials

**DOI:** 10.3390/s16020187

**Published:** 2016-02-02

**Authors:** Catalin-Maricel Jureschi, Jorge Linares, Ayoub Boulmaali, Pierre Richard Dahoo, Aurelian Rotaru, Yann Garcia

**Affiliations:** 1 Faculty of Electrical Engineering and Computer Science & Research Center MANSiD, Stefan cel Mare University, Suceava 720229, Romania; catalin.jureschi@gmail.com (C.-M.J.); aurelian.rotaru@gmail.com (A.R.); 2 LISV, Université de Versailles Saint-Quentin-en-Yvelines, Université Paris Saclay, Velizy 78140, France; 3 GEMaC, Université de Versailles Saint-Quentin-en-Yvelines, CNRS-UVSQ (UMR 8635), Université Paris Saclay, Versailles Cedex 78035, France; 4 LATMOS, Université de Versailles-Saint-Quentin-en-Yvelines, Sorbonne Universités, CNRS-UMR 8190, Université Paris Saclay, Guyancourt F-78280, France; ayouberes@gmail.com (A.B.); pierre-richard.dahoo@uvsq.fr (P.R.D.); 5 Institute of Condensed Matter and Nanosciences, Molecules, Solids and Reactivity (IMCN/MOST), Université Catholique de Louvain, Place L. Pasteur 1, Louvain-la-Neuve 1348, Belgium

**Keywords:** spin crossover, pressure sensors, sensitive paints, optical detection, smart devices

## Abstract

The possibility of a new design concept for dual spin crossover based sensors for concomitant detection of both temperature and pressure is presented. It is conjectured from numerical results obtained by mean field approximation applied to a Ising-like model that using two different spin crossover compounds containing switching molecules with weak elastic interactions it is possible to simultaneously measure P and T. When the interaction parameters are optimized, the spin transition is gradual and for each spin crossover compounds, both temperature and pressure values being identified from their optical densities. This concept offers great perspectives for smart sensing devices.

## 1. Introduction

The miniaturization of electrical, mechanical or optical components is one of the major topical issues which is driven by new horizons that were recently opened with the advances of quantum mechanics. Development of new multifunctional electronic equipment characterized by low energy consumption, or high information processing power, requires high performance electronic components. A special category of such components is represented by sensors, precisely temperature and pressure sensors. The latter are increasingly used in different industrial sectors and particularly in space, aerospace, aeronautics as well as in nuclear fields [1]. As a result, new materials with high technical specifications and able to be controlled at the atomic level are continuously being developed. For high pressure measurements, the main target is to obtain a technological device that allows the measurement of pressure without needing a secondary standard material [2]. For instance, current research are devoted to the insertion of Sm^3+^ in different types of glasses in order to sense pressure using the fluorescence properties of the cation in different environments [3,4,5,6]. The decrease in the lifetime with an increase in pressure is caused by an increase in the electronic transition probabilities which results from the increased crystal-field strength around Sm^3+^ ions. No structural hysteresis takes place around the Sm^3+^ ions. The method involves changing the bond lengths and bond angles between rare earths ions and ligands by applying pressure as in spin crossover (SCO) compounds. In contrast, the corresponding effect on the reflection properties is sought for rather than on absorption ones in SCO compounds. Another method to measure high pressures is through optical methods, such as piezospectroscopy. This was first applied to monitor pressure in diamond anvil cells [7] by measuring the pressure-induced shifts of luminescence lines, from Ruby (R lines of Cr^3+^ [8]) for which the wavelength shift of the R-lines with pressure was shown to be approximately linear. As discussed in reference [2], this technique applied to Sm:YAG allows the primary pressure fluorescence scale to be achieved by simultaneously using two characterization techniques, X-ray diffraction and Brillouin spectroscopy. Indeed, absolute pressures were obtained by integrating the bulk modulus determined via Brillouin spectroscopy with respect to volumes measured simultaneously by X-ray diffraction. The same technique can be used to measure temperature from the stress-induced shifts of the characteristic R-line peaks present in the emission spectrum of alumina as applied to alumina-epoxy composites to determine the thermal stress distribution of encapsulated microelectronic devices [9]. However, with these techniques, only one parameter, either pressure or temperature, is measured. Thus new compounds are needed.

In this context, spin crossover (SCO) materials, are intensely studied due of their ability to control their spin states at the molecular level but also because they are sensitive to temperature or/and pressure variations. These types of materials might offer the opportunity to create a single component sensor which would be able to simultaneously indicate both temperature and pressure variations. Given that such nanomaterials can be miniaturized, this opens up new ranges of applications, saving time and space. In this work, we propose a new sensor concept with optical detection [10] that is based on two different SCO materials deposited as thin films on a detection device. Indeed, several SCO thin film deposition techniques have been already reported such as soft lithography [11,12,13,14,15,16,17], spin coating [18,19], thermal evaporation [20,21] or lithographically controlled wetting [22,23], which opens new perspectives for sensing applications.

## 2. Method and Tools 

**Figure 1:**
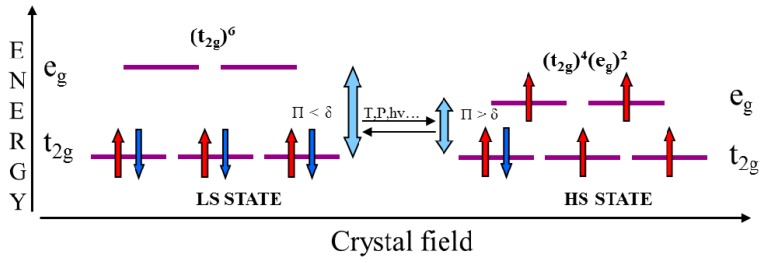
Electronic diagram of the HS and LS states for a Fe(II) ion in an octahedral ligand field.

Spin crossover materials containing an iron(II) cation as central ion in an octahedral configuration are the most studied [24,25,26,27]. The six electrons of the Fe(II) cation can occupy five 3*d* orbitals in two ways depending on the intensity of the ligand field strength (Figure 1):

- In case where the energy gap is higher that the electron spin pairing energy, Δ >> Π, the electrons will occupy the orbitals with lowest energy with the total spin S = 0. This state is called low-spin (LS)
- In the second case when the energy gap is less that the electron spin pairing energy, Δ << Π, the electrons follow the Hund’s rule and will occupy the maximum possible number of orbitals. The total spin will be S = 2 and the state is called high-spin (HS).
- The transition occurs when Δ ≈ Π.

It is interesting to note that the transition between the two stable states can be induced by temperature variation, light irradiation, application of external pressure or magnetic field but also by a chemical adsorption-desorption phenomena [28,29,30,31,32,33]. Once the transition is triggered, the physical and mechanical properties undergo drastic changes. Changes in their color, magnetic state, electrical conductivity or molecular volume are some of these important properties relevant to the concept of a sensor based on SCO materials with multiphysics properties [34,35,36,37,38].

For a complete understanding of these phenomena, both experimental and theoretical works are necessary to determine how SCO compounds are influenced by the action of external factors [39,40,41,42,43]. The temperature and pressure influence on the behavior of a SCO compound are studied in the frame of the Ising-like model. The mean field approximation theory is applied to solve the Hamiltonian of the interaction spins. The Ising-like model, proposed for the first time by Wajnsflasz and Pick [44] uses a fictitious spin operator *σ* which can take the value -1 for the LS state and the value +1 for the HS state with respective degeneracies *g_LS_* and *g_HS_*. Linares *et al.* [45] proposed the explicit introduction of short- and long-range interactions, *J* and *G* respectively in the model. Thus, the Hamiltonian might be written as:

(1) $$H=\frac{\Delta -k_{B}T\ln g}{2}\sum_{i}\sigma_{i}-J\sum_{i,j}\sigma_{i}\sigma_{j}-G\langle \sigma \rangle \sum_{i}\sigma_{i}$$

where *Δ* represents the energy gap between the HS and LS states, $\sum_{i,j}$ is the sum over nearest neighbor spins, *g* = *g_HS_*/*g_LS_* is the degeneracy ratio and *k_B_* is the Boltzmann constant.

The proportion of HS molecules is represented by the HS fraction, *n_HS_*, and is given by:

(2) $$n_{HS}=\frac{\langle \sigma \rangle +1}{2}$$

where 〈σ〉 is the thermal average of σ which can be written as:

(3) $$\langle \sigma \rangle =\tanh\left(\frac{2\Gamma \langle \sigma \rangle +k_{B}T\ln g-\Delta }{2k_{B}T}\right)$$

where Γ=qJ+G was introduced for simplification and *q* is the number of neighbors.

Taking into account the influence of pressure, the energy gap is given by [46]:

(4) Δ=Δ′+pδV

where Δ′=Δ(T,p=0), δV is the volume variation and *p* is the external applied pressure.

In this case the Equation (3) can be rewritten as:

(5) $$\langle \sigma \rangle =\tanh\left(-\frac{\Delta^{\prime }+p\delta V-k_{B}T\ln g-2\Gamma \langle \sigma \rangle }{2k_{B}T}\right)$$

and taking into account the unit used:

(6) $$\langle \sigma \rangle =\tanh\left(-\frac{\frac{\Delta^{\prime }}{k_{B}}+pc\delta V-T\ln g-\frac{2\Gamma \langle \sigma \rangle }{k_{B}}}{2T}\right)$$

where *c* is a constant, c = 0.0724, when the external applied pressure, *p*, is expressed in MPa and the volume variation of the molecule, *δV*, is expressed in Å^3^.

## 3. Results

**Figure 2:**
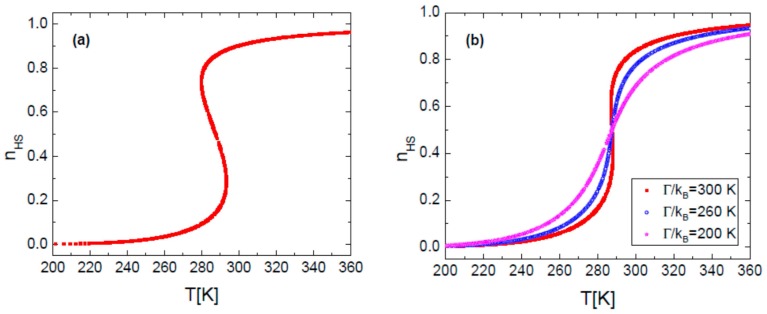
(**a**) Simulated HS fraction n_HS_ *vs.* temperature for Γ/k_B_ = 360 K; (**b**) Simulated HS fraction n_HS_ *vs.* temperature for different values of interaction parameter. The calculation parameters are: Δ’/k_B_ = 1978.6 K, ln(g) = 6.906, δV = 100 Å^3^, p = 0.1 MPa.

**Figure 3:**
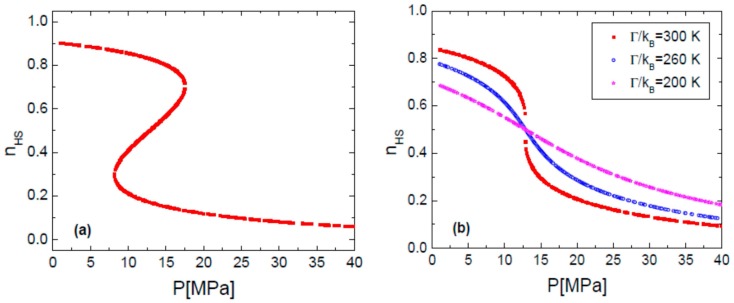
Simulated HS fraction n_HS_ *vs.* pressure for (**a**) Γ/k_B_ = 360 K and (**b**) for different values of Γ; The calculation parameters are: Δ’/k_B_ = 1978.6 K, ln(g) = 6.906, δV = 100 Å^3^, T = 300 K.

It is known that for a large value of the interaction parameter, *Γ*, the spin transition curve is accompanied by an hysteresis loop (Figure 2a and Figure 3a). By decreasing *Γ*, for instance by diluting the SCO compound with diamagnetic species (e.g., Zn(II)), the transition proceeds more gradually and the hysteresis loop can be suppressed. Figure 2b and Figure 3b show different situations, either with fixed *P* at variable *T* or at fixed *T* with variable *P* for different interaction parameters, respectively.

Results plotted in Figure 2b and Figure 3b show that changing the value of the interaction parameter, does not change the transition temperature between spin states, *T_1/2_*. Analyzing Figure 2 and Figure 3, it can be concluded that a gradual transition is requested when both temperature and pressure vary in order to use a SCO compound as a sensor [10]. This means that the compound’s cooperativity in the crystalline state must by very weak. Experimentally, in the last decade several examples of SCO compounds with a gradual transition were reported [47,48,49,50] and in particular within the family of Fe(II) 1D coordination polymers that present a strong optical contrast between spin states [51].

**Figure 4:**
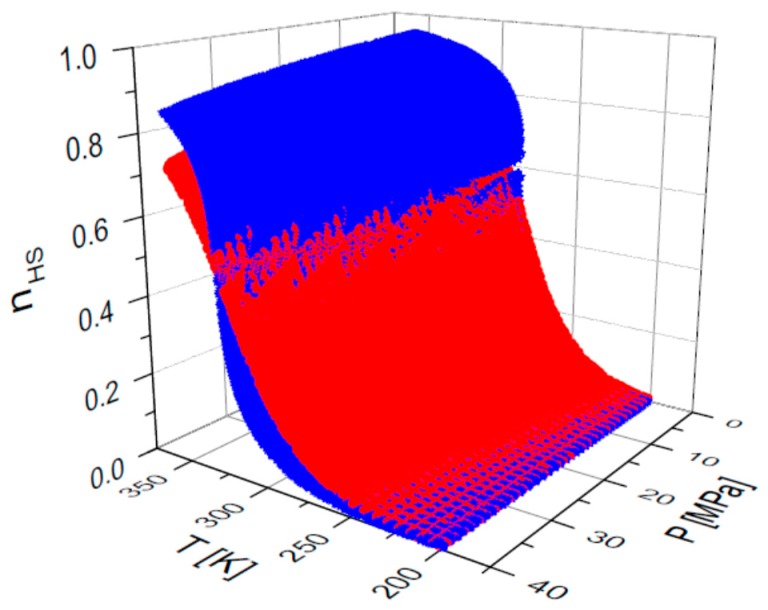
Simulated HS fractions as a function of temperature and pressure variations for two values of the interaction parameter (red—Γ/k_B_ = 160 K and blue—Γ/k_B_ = 300 K). The calculation parameters are: Δ’/k_B_ = 1978.6 K, ln(g) = 6.906, δV = 100 Å^3^.

We have discussed the use of SCO compounds as active components in thermal and pressure sensor devices in a few papers [10,35,52], but did not yet consider the attractive possibility to monitor at the same time both pressure and temperature due to the existence of multiple sets of *T*, *P* solutions that results from the p-T phase diagram (see Figure 4).

On the other hand, by taking into account the behavior of two compounds simultaneously, then only a single temperature and pressure set values are associated to a given set value of HS fraction of the two compounds. The fictitious magnetizations of the two compounds can be written as:

(7) $$\left\{ \begin{array}{l} \langle \sigma_{1}\rangle =\tanh\left(-\frac{\Delta_{1}^{\prime }+pc\delta V-T\ln g_{1}-2\Gamma_{1}\langle \sigma_{1}\rangle }{2T}\right)\\ \langle \sigma_{2}\rangle =\tanh\left(-\frac{\Delta_{2}^{\prime }+pc\delta V-T\ln g_{2}-2\Gamma_{2}\langle \sigma_{2}\rangle }{2T}\right) \end{array}\right.$$

From Equation (7), the following expressions for *T* and *p* can be derived:

(8) $$\left\{ \begin{array}{l} T=\frac{2\Gamma_{1}\langle \sigma_{1}\rangle -\Delta_{1}^{\prime }-2\Gamma_{2}\langle \sigma_{2}\rangle +\Delta_{2}^{\prime }}{2\;\tanh^{-1}\langle \sigma_{1}\rangle -\ln g_{1}-2\;\tanh^{-1}\langle \sigma_{2}\rangle +\ln g_{2}}\\ p=\frac{\left(2\Gamma_{1}\langle \sigma_{1}\rangle -\Delta_{1}^{\prime }\right)*\left(2\;\tanh^{-1}\langle \sigma_{1}\rangle -\ln g_{1}-2\;\tanh^{-1}\langle \sigma_{2}\rangle +\ln g_{2}\right)-\left(2\Gamma_{1}\langle \sigma_{1}\rangle -\Delta_{1}^{\prime }-2\Gamma_{2}\langle \sigma_{2}\rangle +\Delta_{2}^{\prime }\right)*\left(2\;\tanh^{-1}\langle \sigma_{1}\rangle -\ln g_{1}\right)}{c\delta V*\left(2\;\tanh^{-1}\langle \sigma_{1}\rangle -\ln g_{1}-2\;\tanh^{-1}\langle \sigma_{2}\rangle +\ln g_{2}\right)} \end{array}\right.$$

**Figure 5:**
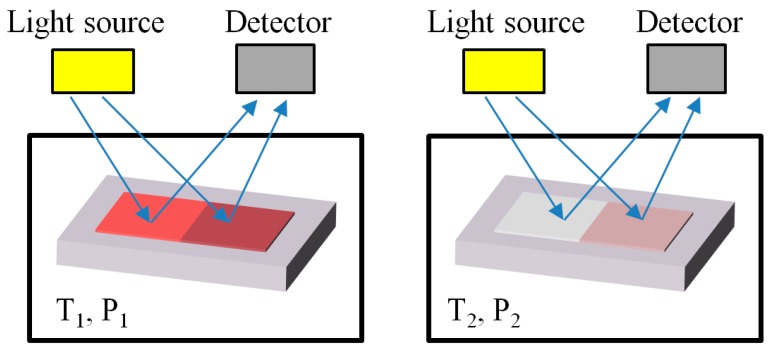
Principle of measuring simultaneously temperature and pressure using two SCO compounds with optical reflectivity detection.

From the system of Equation (8) we can find simultaneously both values of temperature and pressure knowing the values of <σ_1_> and <σ_2_>. These values can be obtained by identifying the optical densities of each complex. The projected device must be initially calibrated using their optical densities. Compounds that could be adapted to this device are: (1) [Fe(PM-A)_2_(NCS)_2_] (PM-A = *N*-(2′-pyridylmethylene)-aniline) [53] with a temperature range between 50 K and 300 K and a pressure range between 0.1 MPa and 1 GPa; (2) [Fe(dpa)(NCS)_2_]_2_bpym (dpa = 2,2′-dipyridylamine; bpym = 2,2′-bipyrimidine) [54] with a temperature range 10 K–350 K; (3) [Fe(C_8_-trz)_3_](tos)_2_∙H_2_O (C_8_-trz = 3,5-dioctyloxy-*N-*4*H*-1,2,4-triazol-4-ylbenzamide; tos = tosylate) with a temperature range between 25 K and 250 K [55]. Practically, our concept can be described as follows. A monochromatic light source (green light = 540 nm) is sent to both compounds. The “scattered” or reflected light by the compounds are directed on the detector (see Figure 5). The detector, calibrated previously, will assign the <σ_1_> and <σ_2_> that corresponds to the lights coming from both compounds. The temperature and pressure values are obtained by replacing the <σ_1_> and <σ_2_> in Equation (8). The challenge for chemists will be to synthesize SCO compounds with a good optical contrast and which will be thermally, pressure and time stable. In other words, to deliver samples which keep the same color over time at a defined temperature and pressure set. This objective looks not too far to be achieved considering recent developments in the SCO field.

## 4. Conclusions

In this work we have presented a novel concept for spin crossover-based sensors that allow the concomitant detection of both temperature and pressure. This new approach is based on the use of two complexes exhibiting gradual spin transitions that leads to the identification of a single set of (P-T) values for a single set of optical densities of each complex. Thus, a simple color identifier is sufficient to detect and measure the variation of both temperature and pressure, opening new perspectives in the multifunctional sensors field. As far as the necessary apparatus needed to monitor temperature and pressure changes, the one proposed here are comparatively much simpler to implement than the existing ones with sophisticated X-Ray diffractometers and apparatuses needed for Brillouin spectroscopy. It consists of standard laser source, detector and associated computer for data analysis. Signal to noise ratio is much better with reflected laser beam than with detection of laser induced fluorescence or luminescence.
